# HIV Associated Neurocognitive Disorders Subsidence Through Citalopram Addition in Anti-retroviral Therapy (HANDS-CARE): A Concept Note

**DOI:** 10.3389/fneur.2021.658705

**Published:** 2021-07-26

**Authors:** Akin Ojagbemi

**Affiliations:** Department of Psychiatry, World Health Organization Collaborating Centre for Research and Training in Mental Health, Substance Abuse and Neuroscience, College of Medicine, University of Ibadan, Ibadan, Nigeria

**Keywords:** acquired immunodeficiency syndrome, HIV associated dementia, antidepressants, low and middle income countries, HIV and AIDS

## Abstract

There is a pressing need to effectively manage HIV Associated Neurocognitive Disorders (HAND) in sub-Saharan Africa (SSA) where the burden is among the highest in the world. Contemporary approaches based on the use of Highly Active Antiretroviral Therapy (HAART) alone are inadequate interventions for HAND, especially in SSA where there is limited availability of the required combinations of HAART for effective central nervous system penetration and where many currently prescribed agents, including efavirenz, have neurotoxicity as a major drawback. This article reviews data supporting the rationale for additive citalopram in antiretroviral therapy as a latent approach to abate HAND. It proposes the conduct of a HIV Associated Neurocognitive Disorders Subsidence through Citalopram addition in *A*nti-*Re*troviral therapy (HANDS-CARE) pilot feasibility trial (RCT) to assess whether the adjunctive use of citalopram, a widely prescribed serotonergic antidepressant, will lead to a meaningful improvement in neurocognitive functioning and quality of life in patients with HAND who are receiving HAART. A preliminarily feasible and efficacy-suggesting HANDS-CARE trial could generate statistical, clinical and operational data necessary to design and conduct a future definitive RCT. If successful, this intervention will be applicable to resource-limited settings as well as developed countries. Effective management of HAND will improve the quality of life of HIV patients, and reduce the cost of managing the disease.

## Introduction

Countries in Sub-Saharan Africa (SSA) account for two-thirds of all People Living with Human Immunodeficiency Virus (HIV) and Acquired Immune Deficiency Syndrome (PLWHA) (1, 2). Previously considered a death sentence, the introduction of increasingly potent combinations of Highly Active Antiretroviral Therapy (HAART) has prolonged survival and reduced morbidity (3, 4). However, this success has not translated to reduction in the global burden of some complications of HIV infection, for example, dementia and other neurocognitive disorders (5, 6).

HIV Associated Neurocognitive Disorders (HAND) are multifactorial complications resulting from the direct affectation of the central nervous system (CNS) by HIV (7). It occurs on a spectrum that includes HIV associated asymptomatic neurocognitive impairment (ANI), HIV associated mild neurocognitive disorder (MND) and HIV associated dementia (HAD) (5), and has been reported at every stage of HIV/AIDS, including in aviremic treated patients and those with minimal immune-suppression (8). The occurrence of HAND has been linked to functional limitation and poor quality of life (9), which in turn increases dependence and suffering in affected individuals. It is also associated with reduced adherence to HAART (9), thus compromising the effectiveness of HAART, and increasing mortality and the risk of HIV transmission. The prevalence among PLWHA in high income countries may reach up to 50% (9). However, rates of well-over 60% have been frequently reported in some African populations (10).

### The Burden of HAND in Sub-Saharan Africa

About 11.3 million HIV/AIDS patients in SSA are reported to be affected by HAND (10, 11), with approximately half of those affected reported to have either symptomatic MND or HAD (12). Given suggestions that the above estimates are conservative (13), the burden of HAND in SSA is likely to rank among the highest of any region in the World.

There are several reasons for the high rates of HAND in sub-Saharan Africa. Firstly, the region has the highest HIV/AIDS burden in the world (2). Secondly, patients in many parts of SSA frequently present with relatively advanced infection characterized by severe immune-suppression (14), a situation which has been shown to directly predispose PLWHA to HAND (9). Thirdly, while HAART is the main approach to the management of HAND (15), the available antiretroviral agents in most of SSA are limited. Notably, efavirenz, one of the most commonly prescribed agents in the region (8), has neurotoxicity as a major drawback.

A fourth reason for an escalation of the burden of HAND in SSA a is major depression. This psychiatric disorder is intrinsic to the process of HIV/AIDS and HAND (16, 17), including through the reduction of adherence to HAART (18). Major depression has a fairly increased and stable prevalence at every point in the spectrum of the disease (at risk individuals, asymptomatic infected, AIDS syndrome, and HAND syndrome) (16, 17). Evidence suggests that major depression may sometimes be the first symptom of HAND, or may confound its diagnosis and outcome (16, 17). Major depression may also be more prevalent in socio-economically disadvantaged settings globally (19), and because of the extensive overlap between depression symptoms and those of HIV/AIDS and HAND, the psychiatric disorder may go unrecognized and untreated in these settings (20).

## Contemporary and Latent Approaches to the Management of HAND

Some of the commonly proposed strategies for the management of HAND include the early initiation of HAART (15), and the use of combinations of HAART which, by possessing higher assumed CNS Penetrating Effectiveness (CPE) scores (21, 22), are thought to have an advantage of better CNS penetration. The use of higher doses of available agents has also been suggested as a feasible strategy in settings, such as those in SSA where there is only a limited combination of HAART (23).

The rationale for the above suggested strategies is based on the observation that, whereas the blood brain barrier may hinder CNS penetration of HAART (24), a higher concentration of HAART is required for the inhibition of macrophage replication within the brain when compared with the concentration required for T- lymphocytes (25). Despite this supposition, the outlined strategies for the management of HAND in contemporary practice have important limitations. First, therapeutic failures have been widely observed with the use of HAART for the treatment of HAND (9). Some reports have also suggested that up to a quarter of PLWHA experience new neurocognitive impairments after initiation of HAART (26). Second, it remains unclear whether the use of antiretroviral agents with higher CPE scores confer actual and unique advantages or whether early introduction of HAART benefit HAND prospectively in the medium to longer term (27). Given these limitations, it would appear that HAART alone is an inadequate intervention for HAND.

A third limitation of the use of HAART for the management of HAND is the question of potential neurotoxicity of many of the available combinations, including efavirenz, a widely prescribed antiretroviral drug globally. These problems are amplified in resource poor settings like Nigeria, where HAART initiation is often delayed and the available antiretroviral agents are limited. Therefore, there is an urgent need to discover interventions that can prevent or mitigate HAND globally, but especially in resource limited SSA settings like Nigeria.

## The Role of Antidepressants in HIV/AIDS and HAND

Emerging evidence from *in-vitro*, human plasma, and cerebrospinal fluid (CSF) studies suggest that serotonergic antidepressants may modulate the pathogenesis of HIV/AIDS and HAND in several possible ways. First, serotonergic antidepressants are known to reduce HIV viral replication in both the CSF and plasma (28, 29). Secondly, serotonergic antidepressants, especially citalopram, have been shown to prevent CNS macrophage infection in *in-vitro* studies (28, 30). Thirdly, serotonergic antidepressants are known to boost the activity of Natural Killer (NK) cells (31). Natural Killer (NK) cells play important roles in the host defense against the HIV virus by producing an HIV suppressive factor which, in turn, prevents viral entry into healthy cells (31). In addition, these cells have been shown to bring about both cytolytic and non-cytolytic destruction of infected cells (32). An additional mechanism is through the immune boosting potentials of serotonergic antidepressants. This is via the immune-modulatory properties of prolactin. The secretion of this hormone is increased in PLWHA treated with some classes of serotonergic antidepressants (33). The secretion of prolactin in patients using serotonergic antidepressants occurs through the inhibition of dopamine, a neurotransmitter that inhibits prolactin secretion (34). It is noteworthy that the above listed effects of serotonergic antidepressants are independent of the depression status of PLWHA (29).

Key information synthesized from 4 previous trials of antidepressants for HAND is summarized in Table 1. Of the 4 previous trials, 1 was non-randomized (36). The remaining 3 were randomized controlled trials (RCT) (35, 37, 38). All trials included adult HIV seropositive patients with evidence of cognitive impairment and a stable antiretroviral regimen for between 6 and 12 weeks. A RCT of oral paroxetine, the only prior study of a selective serotonine re-uptake inhibitor (SSRI) to reduce HAND (37), demonstrated improvement in total and item-specific neuropsychological battery scores. An open label trial of oral lithium (36) also demonstrated improvement in both total and item-specific neuropsychological battery scores. Two RCTs of monoamine oxidase inhibitors (35, 38) demonstrated improvements in some items, but not total score, of a neuropsychological test battery.

**Table 1:** Previous trials of antidepressants for HAND.

**References**	**Study design**	**Number of participants**	**Intervention**	**Duration**	**Control intervention**	**Outcome measures**	**Measure of effect**	**Effect**
Dana consortium	Randomized double-blind	36	Oral deprenyl monoamine oxidase inhibitor (MAOI)	10 weeks	Placebo	Neuropsychological battery test scores	Analyses of variance	1. RAVLT delayed recall 2. Cal CaP reaction time **Authors' conclusion:** deprenyl treatment is associated with cognitive improvement in subjects with mild HIV-associated cognitive impairment
Sacktor et al. (35)	Randomized double-blind	14	Transdermal selegiline MAOI	10 weeks	Placebo	Neuropsychological battery test scores	Analyses of covariance	1. RAVLT delayed recall 2. Grooved Pegboard test **Authors' conclusion:** Selegilline resulted in improved performance in two neuropsychological tests
Letendre et al. (36)	Single-arm, open-label	8	Oral Lithium	12 weeks	Not applicable	Neuropsychological test battery	Paired t-test	Global deficit scores **Authors' conclusion:** Lithium resulted in improved neuropsychological performance
Sacktor et al. (37)	Randomized double-blind	22	Oral paroxetin	24 weeks	Placebo	Neuropsychological test battery	Generalized linear regression models	1.Summary score 2. Cal CAP reaction time, 2. Timed Gait **Authors' conclusion**: Paroxetine was associated with improvement in a summary neuropsychological test measure and in several neuropsychological tests

*RAVLT, Rey Auditory Verbal Learning Test; Cal Cap, California Computerized Assessment Package reaction time test*.

### Specific Effect of Serotonergic Antidepressants in Abating HAND

The specific mechanism that underlies the effectiveness of serotonergic antidepressants in ameliorating HAND is yet unclear. However, as HAND is a complication resulting from the direct affectation of the CNS by HIV (7), it would be feasible that the use of an agent that can potentially prevent viral replication (in the CSF and plasma), healthy macrophage infection, and cause the destruction of infected cells may be a latent strategy to prevent and treat HAND.

Other hypotheses may include:

1. As both HAND and depression are sub-cortical complications of HIV/AIDS (39), it is feasible that both have overlapping origins.
2. Serotonergic antidepressants may potentiate the effect of HAART, which is currently the mainstay in the management of HAND, by increasing uptake of antiretroviral molecules in PLWHA (29).
3. HIV infected persons who are on serotonergic antidepressants, but not on HAART, have been shown to be less likely to have detectable CNS RNA (30, 40), thus, it would be reasonable to postulate that this class of antidepressants have an independent effect of reducing CNS RNA levels in PLWHA.

In addition to the above listed hypotheses, the particular observation of independent reduction of brain RNA levels in PLWHA by citalopram, sertraline and trazodone (30) would suggest that it is difficult to simply ascribe the benefit of the antidepressants on CNS RNA suppression to the effect of the drugs in improving uptake or adherence to HAART.

### Unique Potential of Citalopram in Abating HAND

Among available serotonergic antidepressants, citalopram is the most attractive to investigate in the effort to abate HAND. This is because, apart from the benefits listed above for the broader categories of serotonergic antidepressants, and which have also been specifically demonstrated for citalopram (30, 41), the molecule may have the lowest interaction with available HAART.

Certain individual components of the commonly used HAART, such as Protease Inhibitors (PI) and the Non-Nucleoside Reverse Transcriptase Inhibitors (NNRTI), interact with the Cytochrome P450 by either inhibiting (e.g., ritonavir) or inducing (e.g., nevirapine and efavirenz) the enzyme system in the liver (42). These interactions result in variability in the bio-availability of serotonergic antidepressants metabolized via the same enzyme system (Cytochrome P450), and result in serotonergic toxicity in some cases (40, 42). The interaction between SSRI and Cytochrome P450 occur mostly due to the 2D6 and 3A4 iso-enzymes of the cytochrome system (43–45). However, not all SSRIs are metabolized by both iso-enzymes. For instance, while drugs such as fluoxetin, fluvoxamine, and paroxetine are primarily metabolized through the 2D6 iso-enzyme, sertraline is metabolized through 3A4 (43, 45). Using any of these drugs in combination with a PI, for example, may still result in an increase in their concentration and hence toxicity (42).

Citalopram and its S-enantiomer, escitalopram, are not significantly affected by the action of PIs on the 2D6 and 3A4 isoenzymes (42, 45, 46). Citalopram in particular, is metabolized by a different iso-enzyme of the cytochrome P450 system, the 2C19 (43). Among the more commonly used HAART, only efavirenz has been demonstrated, but not consistently (47), to have a modest to minimal, substrate dependent, mixed inhibition and inducer activity on 2C19 (47, 48). Given the above evidence, it would appear that the 2C19 iso-enzyme is mostly un-affected by a majority of available anti-retroviral agents. For this reason, citalopram and escitalopram are now regarded as the two SSRIs of first choice in patients with HIV/AIDS (48). Apart from this advantage of citalopram (i.e., metabolism is dependent on the 2C19 iso-enzyme of the cytochrome system, and not 2D6 and 3A4), the molecule may also be better tolerated in HIV/AIDS patients since it lacks many of the side effects of the older antidepressants (46, 49).

## A “HANDS-CARE” Strategy to Reduce HIV Associated Neurocognitive Disorders

The potential effectiveness of adjunctive citalopram in the treatment of HAND has not been systematically investigated in a randomized controlled trial (RCT). Proposed in this review is a pilot phase 2 (proof of concept) pragmatic single center RCT, HIV Associated Neurocognitive Disorders Subsidence through Citalopram addition in Anti-Retroviral therapy (HANDS-CARE). The proposed proof of concept HANDS-CARE study could generate statistical, clinical and operational data necessary to design and conduct a future definitive RCT assessing whether the adjunctive use of citalopram, a widely prescribed serotonergic antidepressant, will lead to a meaningful improvement in neurocognitive functioning and quality of life in patients with HIV associated neurocognitive impairment while receiving HAART in Nigeria. If successful, this intervention will be applicable to Nigeria and other resource-limited settings and exportable to more-resourced developed countries.

### Importance and Rationale

Nigeria is the most populous country in Africa (About 200 million people) and may have the largest number of persons with HIV/AIDS (50). The burden of HAND in Nigeria is likely to rank among the highest in the world (3 million people in 2010) (51, 52) because of the massive HIV/AIDS burden, late presentation of patients with relatively advanced infection, and a limited range of available combinations of anti-retroviral agents. Therefore, the need to effectively manage HAND is especially pressing in the country setting. Accordingly, the data generated from the proposed HANDS-CARE proof of concept study will broadly influence the care of HIV patients and shape the design of future trials on HAND and other neuropsychological conditions. The normative data from the proposed study could also be used for future research and training well into the foreseeable future.

### Specific Questions That Could Be Addressed by a HANDS-CARE Proof of Concept Study

This proposed HANDS-CARE proof of concept study could address the following questions:

1. What are the age, gender and education specific norms for neuropsychological functioning in the Nigerian sample of PLWHA?
2. What are the means, standard deviations, and effect sizes, derived from a Standard neuropsychological battery, necessary to determine the appropriate sample size for a definitive RCT?
3. What magnitude of change in relevant single or composite neuropsychological measures is clinically significant (e.g., linked to improvement or worsening of quality of life?)
4. What is the magnitude of change in neuropsychological measures that can be expected from citalopram in HAND patients? And whether this is likely to achieve the threshold of clinical relevance determined in Question 3 above?
5. What are the logistical, programmatic, and operational needs to implement the anticipated definitive citalopram RCT in Nigeria?

### How the Answers to These Questions Will Be Useful in Informing the Design of a Fully Powered RCT

The datasets outlined in the questions above and the operational preparedness for a large HANDS-CARE RCT is currently unavailable in Nigeria. The importance of each question proposed to the design of a future RCT is as follows:

1. *Ascertainment of age, gender and education specific norms for neuropsychological functioning in the Nigerian sample of PLWHA*: This could allow for a precisely defined cognitive impairment by the local norms. Without this foundational dataset, it will be impossible to conduct a rigorous neuropsychological evaluation of HAND in Nigeria. Previous Nigerian studies of cognitive functioning in HIV/AIDS have either relied on neuropsychological tests which were not previously validated for the setting (53), or measures and norms validated for Alzheimer's dementia (54). Data from outside Nigeria are also scant and, more importantly, are suboptimal given important cultural differences and variable literacy levels. A critical advantage of the HANDS-CARE pilot study, therefore, is that it could use measures and tools that are culturally enriched and adapted to the Nigerian context and presented in English and Yoruba, the local language of over 50 million people in South-Western Nigeria.
2. *Ascertainment of the means, standard deviations, and effect sizes, derived from a standard neuropsychological battery*: This is required to determine the sample size that should power a robust larger multicenter HANDS-CARE RCT.
3. *Ascertainment of the magnitude of change in relevant single or composite neuropsychological measures that will be clinically significant*: This data could be essential to assign clinical meaning to neuropsychological changes caused by any specific HAND intervention. The data could also help determine the sample size to appropriately power a definitive HANDS-CARE RCT, and other future studies. The answer to this question is currently unknown.
4. *Ascertainment of the magnitude of change in neuropsychological measures that could be expected from citalopram in patients with HAND*: The information could help determine whether conducting a definitive large citalopram study is a worthwhile venture. A positive result could justify a large HANDS-CARE RCT, while a negative result will save tremendous resources from being committed to a potentially futile large RCT.
5. *Logistical, programmatic, and operational experience gained from the pilot RCT*: Considerations here could include strategies for subject recruitment, research personnel training, and the development of clinical research forms.

## Potential HANDS-CARE Proof of Concept Study Design

A few details to a potential HANDS-CARE proof of concept study with possible participants and procedure are briefly outlined below. An innovative study design could be utilized in two phases;

1. *A cross-sectional study to address the following questions:*
  (a) What are the age, gender and education specific norms for neuropsychological functioning in the Nigerian sample of PLWHA?
  (b) What are the means, standard deviations, and effect sizes, derived from a Standard neuropsychological battery, necessary to determine the appropriate sample size for a definitive RCT?
  (c) What magnitude of change in relevant single or composite neuropsychological measures is clinically significant, e.g., linked to improvement or worsening of quality of life?
2. *A feasibility 24 weeks double blind RCT of a flexible-dose, 20–40 mg/day of citalopram plus HAART, vs. standard treatment of HIV/AIDS with HAART alone*. This phase will address the following questions:
  (a) What is the magnitude of change in neuropsychological measures that can be expected from citalopram in HAND patients? And whether this is likely to achieve the threshold of clinical relevance determined in Question 3 above?
  (b) What are the logistical, programmatic, and operational needs to implement the anticipated definitive citalopram RCT in Nigeria?

The total duration of the HANDS-CARE pilot project could be 24 months (3 months to set up logistics and ethics, 6 months cross-sectional study participants recruitment, evaluation and data analyses, 12 months RCT participants recruitment and follow-up, and 3 months data analyses and dissemination).

### Proposed Approach to HANDS-CARE Phase I

A potential cross-sectional study.

*Potential target population*: We could enroll adult (≥18 years old) HIV seronegative subjects from the general outpatient clinic.

*Overview:* Neurologically healthy, HIV seronegative participants could have a one-time comprehensive neuropsychological assessment. They could be screened for good health using a full physical and neurological examination, a HIV serostatus examination, a detailed history, and assessments of functional status.

*Potential inclusion criteria*:

1. Subjects≥ 18 years of age,
2. Ascertained to be neurologically healthy after a neurological examination, a detailed history, and assessments of functional status.
3. Ascertained to be HIV-seronegative after a HIV serostatus examination

*Potential estimate of sample size:* Eight strata could be utilized; defined by sex (2 levels), age (2 levels), and years of education (2 levels). Based on a previous calculation using the t-distribution (55), it is determined that enrolling 30 participants per stratum could result in a width of 0.746 standard deviation for the 95% Confidence interval of the stratum specific mean, thus we could enroll a total of 240 healthy participants for the eight strata.

*Potential measurements:* Study participants could be administered neuropsychological tests covering the five domains stipulated in the National Institute of Mental Health-sponsored diagnostic (Frascati) criteria (5): verbal fluency (semantic verbal fluency test), executive function (WAIS-R digit symbol test), speed of information processing (Color trails test), verbal learning (Hopkins verbal learning test-revised), and motor speed (Timed gait and grooved pegboard tests). Neuropsychological testing could also include the International HIV dementia scale.

An assessment of activities of daily living, quality of life, and depression could also be included. Validated (in the proposed study population) measures such as the Barthel index, W.H.O quality of life brief version (WHOQOL-BREF), and 9-item patient health questionnaire (PHQ-9), respectively, could be used for these assessments. All test instruments could be translated to the local Yoruba language in Nigeria so that subjects who are Yoruba-only speakers can be assessed in the Yoruba language.

### Proposed HANDS-CARE Phase 2

A potential feasibility 24 weeks double blind RCT of a flexible-dose, 20–40 mg/day of citalopram plus HAART, vs. standard treatment of HIV/AIDS with HAART alone.

*Target population:* we could enroll subjects who are at least 18 years old, confirmed to be HIV-1 infected, and have been suppressed on efavirenz based HAART (plasma 20 copies/mL) for 3 months.

*Potential inclusion criteria:*

1. Adult HIV/AIDS patients
2. Suppressed on HAART for a minimum period of 3 months
3. Have no evidence of clinical depression or other major mental, physical or neurological co-morbidities,
4. Meets Fracati criteria for HAND (5), defined according to the population specific norms (established in phase I), could be enrolled.

*Potential randomization scheme:* HANDS-CARE phase 2 participants could be randomly assigned to intervention and standard treatment groups in a 1:1 ratio. A total of 12 participants (six in each arm) could be enrolled.

*Proposed measurements:* HANDS-CARE phase 2 participants could be assessed at baseline, using the phase I neuropsychological measures and norms, followed up at 4 weeks for the presence of emergent adverse events (A.E). Repeat neuropsychological and A.E evaluations could be conducted at weeks 12 and 24.

*Primary endpoint for efficacy*: This could be a statistically significant improvement from baseline to week 24 (for the citalopram arm) in the composite neuropsychological test score.

*Secondary end points:* Could be a statistically significant change in individual neuropsychological scores. Additional measures could include a change in CD4 count, HIV viral load, adherence to HAART, functioning, weight gain, depression, and quality of life. Safety end points could be the frequency and nature of A.E and retention rate in study.

*Potential mediators of outcome:* Could include demographic characteristics, years of formal education, income, nadir CD4 count, hepatitis C serostatus, alcohol and psychoactive substance use, history of hypertension diabetes or stroke, previous history of depression, baseline severity of cognitive impairment and functional disability, adherence to regimen, presence of mild/ sub-threshold depression symptoms at baseline, insomnia, and neuropathic pain. These variables will be assessed and controlled for.

## Conclusion

Trials evaluating the effectiveness of citalopram as a cheap, safe and readily available adjunctive treatment for HAND are currently needed, and will be especially applicable to resource-poor settings, such as those in SSA, where there is a limited availability of the required variety of HAART combinations with sufficient CNS penetrating effectiveness to target neuro-cognitive complications. Efficient management of HAND will improve the quality of life of HIV patients, and reduce the cost of management of HIV/AIDS in both resource poor and rich settings.

## Data Availability Statement

The original contributions presented in the study are included in the article/supplementary material, further inquiries can be directed to the corresponding author/s.

## Author Contributions

AO conceived the idea and study design, prepared the materials, wrote and approved the manuscript drafts.

## Conflict of Interest

The author declares that the research was conducted in the absence of any commercial or financial relationships that could be construed as a potential conflict of interest.

## Publisher's Note

All claims expressed in this article are solely those of the authors and do not necessarily represent those of their affiliated organizations, or those of the publisher, the editors and the reviewers. Any product that may be evaluated in this article, or claim that may be made by its manufacturer, is not guaranteed or endorsed by the publisher.
